# Oral Antithrombotic Medication Is Associated with Improved Visual Outcomes in Eyes with Submacular Hemorrhage from Wet Age-Related Macular Degeneration

**DOI:** 10.1016/j.xops.2025.100796

**Published:** 2025-04-14

**Authors:** Hemal P. Patel, Cason B. Robbins, Jamie J. Karl, Peter Weng, Lejla Vajzovic, Sharon Fekrat

**Affiliations:** Department of Ophthalmology, Duke University School of Medicine, Durham, North Carolina

**Keywords:** Submacular hemorrhage, Antithrombotics, Macular degeneration, Anticoagulants, Platelet aggregation inhibitors

## Abstract

**Purpose:**

To determine differences in visual acuity (VA) outcomes in eyes of patients on antithrombotics (antiplatelet agents and anticoagulants) that develop submacular hemorrhage (SMH) compared with eyes of patients who are not on antithrombotics and develop SMH.

**Design:**

A retrospective study of patients presenting with fovea-involving SMH due to wet age-related macular degeneration over an 8-year period who had ≥6 months of follow-up.

**Subjects:**

Demographics, VA at presentation and final follow-up after SMH management, and history of any antithrombotic therapy were collected. Patients were grouped based on whether they were on anticoagulants (direct oral anticoagulants or warfarin), antiplatelet agents (P2Y12 inhibitors, aspirin, or both), or neither.

**Methods:**

Multivariate generalized estimating equations were used for statistical analysis.

**Main Outcome Measures:**

The difference between VA at presentation with SMH and VA at final follow-up was compared between patients taking oral anticoagulants or antiplatelet agents and those not on any antithrombotic agent.

**Results:**

Seventy-seven eyes of 74 patients were included. Twenty were on oral anticoagulants, 38 were on antiplatelet agents, and 22 were on neither. After controlling for age, sex, post-SMH cataract surgery, time to presentation, treatments received, initial VA, and follow-up duration, patients taking oral anticoagulants had greater improvement in VA at final follow-up compared with patients who were not taking a concurrent antithrombotic agent (*P* = 0.001); however, patients on oral antiplatelets did not (*P* = 0.09). After additionally controlling for the initial size and thickness of SMH, patients taking oral anticoagulants and patients taking oral antiplatelets had greater improvement in VA at final follow-up compared with patients who were not taking an antithrombotic (*P* = 0.002 and *P* < 0.001, respectively).

**Conclusions:**

Patients taking oral anticoagulants who then develop an SMH may have better long-term VA outcomes than those who were not. However, this effect was not seen in patients taking oral antiplatelet medications. This suggests that baseline anticoagulants may be associated with improved VA outcomes in eyes that develop an SMH. When controlling for size and thickness of SMH, patients taking oral anticoagulants and those taking oral antiplatelets had better long-term VA outcomes than those who were not, suggesting a potential mitigating effect of oral antithrombotics.

**Financial Disclosure(s):**

Proprietary or commercial disclosure may be found in the Footnotes and Disclosures at the end of this article.

Submacular hemorrhage (SMH) may occur in eyes with wet age-related macular degeneration (AMD), even while receiving intravitreal anti-VEGF treatment.^1^ Although improved treatment of macular neovascularization in these eyes has contributed to a reduced incidence of SMH,^2^ the development of SMH can result in poorer visual outcomes.^1^^,^^3^ Thus, understanding risk factors and outcomes associated with SMH may improve the management of patient expectations and counseling.

Individuals with AMD are more likely to have other age-related diseases, such as cardiovascular disease and atrial fibrillation.^4^^,^^5^ These conditions are treated with antithrombotic therapies, including antiplatelet agents, such as aspirin and clopidogrel, and anticoagulants, including direct oral anticoagulants (DOACs) and vitamin K antagonists (e.g., warfarin).^6^ While these therapies are necessary for the management of atrial fibrillation and cardiovascular disease, they may increase the risk of systemic bleeding^7^^,^^8^ and predispose eyes with wet AMD to develop a larger and possibly thicker SMH.^9^, ^10^, ^11^ However, the effect of antithrombotic agents on SMH has not been fully understood in the literature.^9^, ^10^, ^11^, ^12^, ^13^, ^14^, ^15^

Although numerous reviews have investigated the long-term visual consequences of SMH,^1^^,^^16^ to our knowledge, no studies have specifically investigated the effect of antithrombotic agents on the long-term visual consequences of SMH. This retrospective study examined the change in visual acuity (VA) in patients with SMH due to wet AMD who were already taking antithrombotic agents at presentation, compared with patients with SMH who were not taking an antithrombotic agent at presentation.

## Methods

A retrospective review of patients diagnosed with SMH secondary to wet AMD at Duke Eye Center between 2014 and 2023 was performed. Institutional review board approval for this study was obtained from the Duke University Health System Institutional Review Board (Pro0111996). There was no substantial risk to participants for inclusion in the study due to its retrospective nature, and therefore the requirement of informed consent was waived. This study complied with the Health Insurance Portability and Accountability Act of 1996 and the tenets of the Declaration of Helsinki.

Patients were identified using the Duke Enterprise Data Unified Content Explorer (Duke University Health System), a database formed from the clinical information application of the electronic medical record used at Duke Health (Maestro Care). Patients diagnosed with SMH and a known history of wet AMD were included. Images, including fluorescein angiography, ultra-widefield fundus photography, B-scan ultrasonography, and OCT from the date of SMH presentation, were reviewed to confirm the presence of foveal involvement of SMH and that SMH was ≥1 disc diameter (DD) across.^1^ If a patient had bilateral SMH due to wet AMD, both eyes were included in the study.

Patient charts were reviewed to collect demographic data, including age and sex, time to presentation to the Duke Eye Center in days from the development of SMH, and whether a patient was taking antithrombotic agent(s) at the time of presentation with SMH. Charts were then reviewed to determine if the patient was taking aspirin, a P2Y12 inhibitor (clopidogrel, ticlopidine, ticagrelor, prasugrel, and cangrelor), a DOAC (dabigatran, rivaroxaban, apixaban, and edoxaban), or the vitamin K antagonist warfarin. Treatments for SMH that were documented consisted of intravitreal anti-VEGF agents, pneumatic displacement with intravitreal gas with recombinant tissue plasminogen activator (rtPA) administered in a clinic setting, and vitrectomy with subretinal rtPA and intravitreal gas in the operating room. Visual acuity outcome included best-corrected VA at final follow-up measured using the ETDRS chart. The date of final follow-up was also collected to determine how long a patient was followed after the development of SMH, and only patients followed for ≥6 months were included. As some treatments for SMH may increase cataract progression,^17^ whether or not a patient had cataract surgery after the development of SMH was also collected.

Size and thickness of SMH were also considered for secondary analysis where available. The size of the SMH was measured using en face OCT images. Size was determined by measuring the largest diameter across the SMH in DD. Small SMH measured between 1 and 3 DD, medium measured between 4 and 5 DD, and massive SMH were >5 DD and extended beyond the temporal arcades.^1^^,^^18^ Thickness of SMH was determined to be the thickest distance between the inner limiting membrane and the choroid in micrometers (μm) where SMH was identified.^19^ The distance from the inner limiting membrane to the choroid was chosen to account for heterogeneous retinal thickening consistent with intraretinal bleed to best account for the full extent of SMH.

All statistical analyses were completed using StataSE (18.0, StataCorp LLC). Groups were compared using multivariable generalized estimating equations. The generalized estimating equation model was used to account for the inclusion of 2 eyes from the same patient and covariates. There were 4 regression models in this analysis. The dependent variable for all 4 regression models was the difference between VA at final follow-up and VA at presentation with SMH. The ETDRS VA was converted to logarithm of the minimum angle of resolution (logMAR) to linearize VA measurements for the interpretation of linear regression analysis.^20^ The independent variable of interest in the first 2 regression models was whether a patient was taking an anticoagulant at the time of developing SMH. The independent variable in the latter 2 regression models was whether a patient was taking an antiplatelet agent at the time of developing SMH. All 4 regression models contain the common independent variable controls of sex, age in years, treatment with anti-VEGF after development of SMH, treatment with vitrectomy and subretinal rtPA after the development of SMH, treatment with pneumatic displacement and rtPA after the development of SMH, whether a patient had cataract surgery after developing SMH, initial VA, time to presentation, and duration of follow-up years. Time to presentation was controlled for in a stratified approach by comparing patients who presented 7 to 14 days, 15 to 30 days, and >30 days to patients who presented within 7 days of developing SMH. The second and fourth regression models were a subanalysis that additionally controlled for SMH thickness in micrometers and SMH size by comparing medium and massive SMH to small SMH. An alpha of 0.05 was used to determine statistical significance.

## Results

Seventy-seven eyes of 74 patients were eligible for participation (Table 1). The mean age of study participants was 77.2 years with a standard deviation of 9.5, and 29 of 74 (39.2%) patients were male. Treatment for SMH included intravitreal anti-VEGF injections in the clinic for 67 of 77 (87%) eyes, pneumatic displacement with intravitreal rtPA and gas administered in the clinic setting for 8 of 77 (10%) eyes, and vitrectomy with subretinal rtPA and intravitreal gas in the operating room for 37 of 77 (48%) eyes. Twenty of 77 (26%) eyes underwent cataract extraction and intraocular lens placement after SMH, while 43 of the remaining 59 eyes were pseudophakic at the time of SMH. Fourteen of 77 (18%) eyes remained phakic at final follow-up. For treatment with antithrombotic agents, 38 cases (49%) of SMH occurred while a patient was taking oral antiplatelet therapy, while 20 (26%) occurred while a patient was taking anticoagulants. Four occurred when a patient was taking both, and 22 (29%) occurred when a patient was on neither. Of 38 cases involving antiplatelet therapy, 11 (29%) were only on aspirin, 1 (3%) was only on a P2Y12 inhibitor, and 26 (68%) were on dual antiplatelet therapy with both aspirin and P2Y12 inhibitor. Of 20 cases involving anticoagulant therapy, 12 (60%) were on DOACs including apixaban and rivaroxaban, and 8 (40%) were on warfarin.

**Table 1:** Demographic Data

Variables	All Patients (n = 77 Eyes of 74 Patients)∗
Age (yrs)	
Mean (SD)	77.2 (9.46)
Range	(53.8–98.4)
Duration of follow-up (yrs)	
Mean (SD)	3.0 (2.4)
Range	(0.5–9.3)
Sex, male/female	29/45
Treatment	67 anti-VEGF in clinic
	37 vitrectomy
	8 pneumatic displacement in clinic
Cataract surgery after SMH	20
Antiplatelet therapy	N = 38
	11 ASA only
	1 P2Y12 only
	26 DAPT
Anticoagulant therapy	N = 20
	8 on warfarin
	12 on DOACs

ASA = aspirin; DAPT = dual antiplatelet therapy; DOAC = direct oral anticoagulant; SD = standard deviation; SMH = submacular hemorrhage.

∗ Three patients had submacular hemorrhage in both eyes.

Of the patients not on an antithrombotic agent, the mean age was 75.6 years, average time to presentation was 17.8 days, and 3 of 22 (14%) were male. Eighteen of 22 (82%) eyes were treated with intravitreal anti-VEGF injections in the clinic, none had pneumatic displacement with intravitreal rtPA and gas administered in the clinic setting, and 10 of 22 (46%) underwent vitrectomy with subretinal rtPA and intravitreal gas in the operating room. Eight of 22 (36%) eyes subsequently underwent cataract extraction and intraocular lens placement. Mean VA at presentation was 20/640, and mean VA at final follow-up was 20/750. Compared with presentation, VA at final follow-up on average worsened by about 3.5 letters on the ETDRS chart (0.067 logMAR units). Mean follow-up time was 3.20 years, with a standard deviation of 2.86 years (Table 2). Sixteen of 22 eyes had available OCT images at presentation (Table 3). Eight were small SMH (1–3 DD), 1 was medium (4–5 DD), and 7 were massive (>5 DD). Average thickness was 1097.5 μm with a standard deviation of 404.8 μm.

**Table 2:** Demographic Data by Antithrombotic Status

Variables	On Antiplatelets	On Anticoagulants	No Antithrombotic Agent
Number of subjects∗	38	20	22
Age (yrs)			
Mean (SD)	77.0 (8.1)	80.0 (7.6)	75.6 (12.1)
Range	61–93	65–92	53–98
*P* value†	0.61	0.17	
Sex			
Male/female	20/18	8/12	3/19
*P* value	0.003	0.05	
Time to presentation (days)			
Mean (SD)	18.3 (32.0)	9.8 (20.0)	17.8 (25.3)
Range	0–108	0–91	0–97
*P* value	0.95	0.26	
Time to presentation n (%)			
<7 days	22 (57.9)	12 (60)	11 (50)
7–14 days	8 (21.1)	5 (25)	4 (18.2)
15–30 days	2 (5.3)	2 (10)	2 (9.1)
>30 days	6 (15.8)	1 (5)	5 (22.7)
Treatment n (%)			
Pneumatic displacement	4 (10.5)	4 (20)	0 (0)
Vitrectomy	20 (52.6)	8 (40)	10 (45.5)
Anti-VEGF	32 (84.2)	18 (90)	18 (81.8)
Cataract surgery after SMH n	9	2	8
*P* value	0.29	0.05	
VA at presentation	20/800	20/1180	20/640
*P* value	0.61	0.24	
VA final visit	20/430	20/250	20/750
*P* value	0.33	0.11	
Time of follow-up (yrs)	2.82 (2.23)	2.77 (2.19)	3.20 (2.86)
*P* value	0.57	0.32	
Difference between VA at final visit and presentation (logMAR)	−0.27	−0.67	0.067
*P* value	0.21	0.02	-

logMAR = logarithm of the minimum angle of resolution; SD = standard deviation; SMH = submacular hemorrhage; VA = visual acuity.

∗ Three patients were on both.

† *P* values provided for comparison with subjects taking no antithrombotics. For continuous variables, *P* values are provided using 2-sided *t* test. For categorical variables, they are provided using Pearson chi-square.

**Table 3:** Structural Analysis of SMH

Variables	No Antithrombotic Agent	On Antiplatelets	On Anticoagulants	On Both Antiplatelet and Anticoagulant
SMH largest diameter n∗				
1–3 DD	8	12	7	1
4–5 DD	1	5	1	0
>5 DD and beyond vascular arcades	7	13	5	1
*P* value†	0.94			
SMH thickness n	16	29	13	1
Mean thickness in μm (SD)‡	1097.5 (404.8)	1128.6 (390.2)	1131.1 (367.4)	585 (0)
*P* value§				-

DD = disc diameter; ILM = internal limiting membrane; SD = standard deviation; SMH = submacular hemorrhage.

∗ Sixty-one of 77 had available OCT images at presentation. The SMH size was determined using the en face images of the OCT.

† *P* values provided using Fisher exact test.

‡ Fifty-nine of 61 had ample quality OCT images to determine thickness. Thickness was measured from the ILM to the choroid.

§ *P* value provided using chi-squared Bartlett test of equal variance. Because there was only 1 observation in the group taking both antithrombotics, this was not included in this test.

Of the 38 patients on an antiplatelet agent, the mean age was 77.0 years, the average time to presentation was 18.3 days, and 20 of 38 (53%) were male. Patients on antiplatelet agents did not significantly differ from patients not on an antithrombotic agent with regard to age (*P* = 0.61) but did differ by sex (*P* = 0.003). Thirty-two of 38 (84%) were treated with intravitreal anti-VEGF injections in the clinic, 4 of 38 (11%) had pneumatic displacement with intravitreal rtPA and gas administered in the clinic setting, and 20 of 38 (53%) underwent vitrectomy with subretinal rtPA and intravitreal gas in the operating room. Nine of 38 (24%) eyes subsequently underwent cataract extraction and intraocular lens placement. Mean VA at presentation was 20/800, and mean VA at final follow-up was 20/430. Compared with presentation, VA at final follow-up on average improved by about one line on the ETDRS chart (0.27 logMAR units). Mean follow-up time was 2.82 years, with a standard deviation of 2.23 years (Table 2). Thirty of 38 eyes had available OCT images at presentation (Table 3). Twelve were small SMH (1–3 DD), 5 were medium (4–5 DD), and 13 were massive (>5 DD). Of these, thickness was assessable on 29 of 38 images. Average thickness was 1129 μm with a standard deviation of 390 μm.

Of the 20 patients on an anticoagulant, the mean age was 80.0 years, the average time to presentation was 9.8 days, and 8 of 20 (40%) were male. Patients on antiplatelet agents did not significantly differ from patients not on an antithrombotic agent with regard to age (*P* = 0.17) but did differ by sex (*P* = 0.05). Eighteen of 20 (90%) were treated with intravitreal anti-VEGF injections in the clinic, 4 of 20 (25%) had pneumatic displacement with intravitreal rtPA and gas administered in the clinic setting, and 8 of 20 (40%) underwent vitrectomy with subretinal rtPA and intravitreal gas in the operating room. Two of 20 (10%) eyes subsequently underwent cataract extraction and intraocular lens placement. Mean VA at presentation was 20/1180, and mean VA at final follow-up was 20/250. Compared with presentation, VA at final follow-up on average improved by about 3 lines on the ETDRS chart (0.67 logMAR units). Mean follow-up time was 2.77 years, with a standard deviation of 2.19 years (Table 2). Thirteen of 20 eyes had available OCT images at presentation (Table 3). Seven were small SMH (1–3 DD), 1 was medium (4–5 DD), and 5 were massive (>5 DD). Thickness was assessed on these 13 images. Average thickness was 1131 μm with a standard deviation of 367 μm.

There was no significant difference in VA at presentation between patients on an antiplatelet agent at presentation and at final follow-up compared with patients on no antithrombotic (20/800 vs. 20/640 at presentation, *P* = 0.61; 20/430 vs. 20/750 at final follow-up; *P* = 0.33, respectively) (Table 2). There was also no significant difference in VA between patients taking an anticoagulant at presentation and at final follow-up compared with patients not taking an antithrombotic agent (20/1180 vs. 20/640 at presentation, *P* = 0.24; 20/250 vs. 20/750 at final follow-up, *P* = 0.11, respectively).

After controlling for age, sex, time to presentation to the clinic, duration of follow-up, whether a patient had subsequent cataract surgery after SMH, initial VA, type of intervention for SMH (pneumatic displacement or vitrectomy), and treatment with anti-VEGF after SMH, patients taking oral anticoagulants improved by 0.866 logMAR units, or about 4 lines on the ETDRS chart, more at final follow-up than patients not taking an antithrombotic agent (*P* = 0.001). In this model, whether a patient had subsequent cataract surgery after SMH, initial VA, and time followed were significant at the 0.05 level, while the other control variables were not (Table 4, column 1). When additionally controlling for size and thickness of SMH, patients taking oral anticoagulants improved by 0.711 logMAR units, or about 3 lines on the ETDRS chart, more at final follow-up than patients not taking an antithrombotic agent (*P* = 0.002). In this model, SMH size was significant at the 0.05 level, while thickness was not (Table 4, column 2). Full regression outputs for these models are available in Tables S5 and S6 (available at www.ophthalmologyscience.org).

**Table 4:** Regression Results

Variables	(1)	(2)	(3)	(4)
	Difference between Final and Presenting VA	Difference between Final and Presenting VA	Difference between Final and Presenting VA	Difference between Final and Presenting VA
On anticoagulant	−0.866 (0.256)∗	−0.711 (0.225)∗		
On antiplatelet			−0.426 (0.247)‡	−0.692 (0.194)∗
Male sex	0.0373 (0.287)	−0.0438 (0.280)	0.330 (0.256)	0.313 (0.216)
Age	−0.00926 (0.0146)	−0.0165 (0.0122)	0.00883 (0.0138)	−0.00296 (0.00973)
Anti-VEGF	0.336 (0.374)	0.487 (0.346)	0.393 (0.325)	0.246 (0.271)
Vitrectomy	0.431 (0.258)∗	0.312 (0.262)	0.155 (0.255)	−0.131 (0.195)
Pneumatic displacement	−0.0741 (0.457)	−0.218 (0.442)	−0.163 (0.497)	−1.026 (0.381)∗
Cataract surgery after SMH	−1.101 (0.308)∗	−0.924 (0.300)∗	−0.431 (0.273)	−0.890 (0.234)∗
Initial VA	−0.528 (0.163)∗	−0.691 (0.155)∗	−0.570 (0.163)∗	−0.744 (0.155)∗
Time followed	0.0968 (0.0490)†	0.0235 (0.0442)	0.548 (0.0498)	0.0286 (0.0373)
Time to presentation compared with <7 days				
7–14 days	0.468 (0.340)	−0.104 (0.313)	−0.0952 (0.314)	−0.151 (0.229)
15–30 days	0.537 (0.409)	0.473 (0.361)	0.336 (0.481)	0.583 (0.451)
>30 days	−0.139 (0.371)	−0.788 (0.371)†	−0.0201 (0.332)	−0.523 (0.257)†
SMH size compared with 1–3 DD				
3–4 DD		1.517 (0.485)∗		2.200 (0.335)∗
>5 DD		1.451 (0.366)∗		1.737 (0.255)∗
SMH thickness (microns)		−0.000606 (0.000458)		−0.000992 (0.000330)∗
Constant	1.074 (1.340)	2.187 (1.131)‡	−0.206 (1.310)	1.780 (0.990)‡
Number of eyes	42	30	60	46
Number of subjects	40	29	59	45

DD = disc diameter; SMH = submacular hemorrhage; VA = visual acuity.

Regression results provided using a multivariate generalized estimating equation model to account for using both eyes of patients with bilateral SMH. Standard errors are in parentheses. Values represent multivariate regression coefficient with standard errors in parenthesis. *P* values are denoted by footnotes.

∗ *P* < 0.01.

† *P* < 0.05.

‡ *P* < 0.1.

After controlling for age, sex, time to presentation to the clinic, duration of follow-up, whether a patient had subsequent cataract surgery after SMH, initial VA, type of intervention for SMH (pneumatic displacement or vitrectomy), and treatment with anti-VEGF after SMH, patients taking antiplatelet agents improved by an additional 0.426 logMAR units, or about 2 lines on the ETDRS chart, at final follow-up than patients not taking an antithrombotic agent; however, this was not statistically significant (*P* = 0.09). In this model, initial VA was significant at the 0.05 level, while the other control variables were not (Table 4, column 3). When additionally controlling for size and thickness of SMH, patients taking oral antiplatelet agents improved by an additional 0.692 logMAR units, or about 3 lines on the ETDRS chart, at final follow-up than patients not taking an antithrombotic agent (*P* < 0.001). In this model, SMH size and thickness were significant at the 0.05 level (Table 4, column 3). Full regression outputs for these models are available in Tables S7 and S8 (available at www.ophthalmologyscience.org).

## Discussion

Submacular hemorrhage can occur in eyes with wet AMD and typically contributes to poor visual outcomes.^1^ The presence of subretinal blood leads to irreversible damage to the retina within 24 hours by creating a diffusion barrier, mechanical damage, and oxidative toxicity.^21^ Antithrombotics increase systemic bleeding,^7^^,^^8^ and this may increase subretinal blood in SMH that impacts structural and functional outcomes.^9^, ^10^, ^11^ Patients taking anticoagulants and antiplatelet agents had significantly improved VA at final follow-up when compared with patients not taking an antithrombotic agent in this retrospective, single-center clinical study.

Although prior studies have found that antithrombotic agents increase the incidence^9^^,^^10^ and size^9^, ^10^, ^11^ of SMH, only 1 previous study has investigated the role of antithrombotic medication on VA outcomes in eyes that develop SMH.^11^ Weber et al found that there was no significant difference in VA at 1 year between patients taking antithrombotic medication and those who were not. With the mean duration of follow-up in our study being 3 years, our study evaluates VA differences over a longer time period. Additionally, Weber et al did not control for type of intervention (pneumatic displacement or vitrectomy) nor size and thickness of SMH, which can be important mediating factors in the long-term outcomes for eyes that develop SMH. Additionally, our study specifically compared VA outcomes in patients on anticoagulants to controls and VA outcomes in patients on antiplatelet medications to controls. As anticoagulant and antiplatelet medications have different mechanisms of action, our findings add to the current literature by specifically comparing each antithrombotic agent group to controls over a longer follow-up period while also controlling for factors that affect VA outcomes including lens status, type of intervention (pneumatic displacement or vitrectomy), treatment with intravitreal anti-VEGF injection, size and thickness of SMH, and time to presentation to the clinic.

Our findings suggest that the concurrent use of anticoagulant agents at the time of SMH development may be associated with improved longer-term visual outcomes. This finding appears paradoxical, as one would hypothesize that with oral antithrombotic agents, SMH would be larger and thicker, and thus more destructive. However, understanding the pathophysiological effect of SMH in the eye is complex and has been attributed to multiple mechanisms.^1^^,^^3^^,^^22^, ^23^, ^24^, ^25^ One relevant proposed mechanism of damage caused by SMH is that as the blood coagulates and the associated fibrin mesh contracts, shearing of the photoreceptors leads to permanent cell damage and visual loss.^26^ It has been reported that fibrin forms as early as an hour after SMH,^21^ and thus inhibition of the formation of fibrin is a common time-sensitive goal of early treatment in eyes with SMH. This has been traditionally accomplished through the use of intravitreal or subretinal rtPA, which is a serine protease that complexes with fibrin to catalyze the conversion of plasminogen to plasmin that, in turn, cleaves fibrin.^27^ The use of rtPA early in the treatment course of eyes with SMH has been suggested to improve visual outcomes.^28^^,^^29^ Anticoagulants work similarly to rtPA by inhibiting clotting factors upstream of fibrin in the clotting cascade. Specifically, DOACs directly inhibit factor Xa,^30^ and vitamin K antagonists like warfarin inhibit factors II, VII, IX, and X, as well as the regulatory factors protein C and protein S.^31^ By inhibiting the coagulation cascade upstream of fibrin, anticoagulants indirectly inhibit the conversion of fibrinogen to fibrin, which may have a protective effect on photoreceptors by inhibiting the formation of the fibrin mesh hypothesized to cause tractional damage,^26^ thus potentially explaining the significantly greater improvement in VA seen in the eyes of patients on anticoagulants observed in this study. Additionally, antiplatelet agents work by inhibiting the aggregation of platelets.^32^ While they do have some indirect ability to inhibit fibrin formation, they do not have the same direct effect on fibrin as anticoagulants.^33^ This could explain why patients taking antiplatelet agents at the time of SMH formation do not exhibit the same level of VA improvement as those on anticoagulants and why the effect is not significant (*P* = 0.09).

Although inhibition of clot formation may lead to larger SMH, as demonstrated in previous studies,^9^, ^10^, ^11^ our study did not find statistically significant differences in size and thickness of SMH in individuals on antithrombotics and those who were not (Table 3). Nevertheless, our study found that eyes that exhibited medium or massive SMH were significantly more likely to have worse VA at final follow-up than those with small SMH in both the anticoagulant and antiplatelet groups (Table 4, columns 2 and 4). In the analysis comparing eyes of patients on anticoagulants to those on no antithrombotic, eyes that experienced medium and massive SMH hemorrhage worsened significantly at final follow-up compared with eyes that experienced small SMH (1.52 and 1.45 logMAR units, respectively), while the improvement of eyes of patients on anticoagulants in the model was 0.711 logMAR units (Table 4, column 2). Similarly, in the analysis comparing eyes of patients on antiplatelet agents to those on no antithrombotic, eyes that experienced medium and massive SMH hemorrhage also worsened significantly at final follow-up compared with eyes that experienced small SMH (2.2 and 1.73 logMAR units, respectively), while the improvement of eyes of patients on antiplatelets in the model was 0.692 logMAR units (Table 4, column 4). Controlling for this large negative effect on long-term VA associated with larger SMH may explain why the improvement in VA associated with being on an antiplatelet agent becomes statistically significant compared with when not controlling for this effect (0.426 logMAR units, *P* = 0.09 vs. 0.692 logMAR units, *P* < 0.001). These findings suggest that although developing a larger SMH leads to subsequent worsening of VA, being on an anticoagulant or antiplatelet agent at the time of SMH may help to mitigate a portion, but not all of that worsening.

This study has several limitations inherent to its retrospective design. Specifically, because of variable follow-up time points for each patient, it was not possible to standardize follow-up visits, and thus, not all patients were followed for the same duration of time after SMH presentation. Additionally, the use of the ETDRS charts to measure VA in patients with counting fingers or hand motion vision may decrease the reproducibility of these values, especially when converting these values to logMAR. Finally, because different imaging modalities were used for some patients, it was not possible to have an in-depth, qualitative analysis of each SMH beyond size and thickness. Future prospective studies may standardize imaging and follow-up time points to further track how antithrombotic agents may impact SMH and associated visual outcomes.

In patients who develop SMH, the concurrent use of anticoagulant medications was associated with better visual outcomes than in patients not taking any antithrombotic agents. This observation was not present in patients solely taking antiplatelet agents. When controlling for size and thickness of SMH, both the concurrent use of anticoagulant medications and antiplatelet medications were associated with better visual outcomes than patients not taking any antithrombotic agents. These findings suggest that the use of antithrombotics may have a mitigating effect on the worsening of VA caused by large SMH. The inhibition of fibrin formation, especially in patients taking anticoagulants, may translate into less shearing of the photoreceptors and thus better VA outcomes for patients on these agents. As such, future larger-scale investigations may confirm our findings and yield additional insights.
