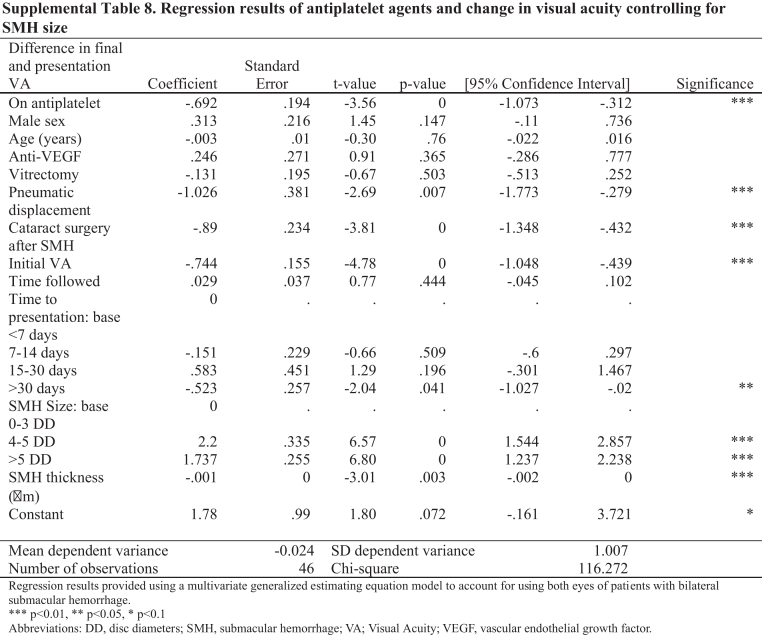# Corrigendum to “Oral Antithrombotic Medication Is Associated with Improved Visual Outcomes in Eyes with Submacular Hemorrhage from Wet Age-Related Macular Degeneration” [Ophthalmology Science. 2025;5:100796]

**DOI:** 10.1016/j.xops.2025.100976

**Published:** 2025-10-10

**Authors:** Hemal P. Patel, Cason B. Robbins, Jamie J. Karl, Peter Weng, Lejla Vajzovic, Sharon Fekrat

**Affiliations:** Department of Ophthalmology, Duke University School of Medicine, Durham, North Carolina

The authors of “Oral antithrombotic medication is associated with improved visual outcomes in eyes with submacular hemorrhage from wet age-related macular degeneration” would like to note the following corrections to their article.

In Table 4, the 14th row of the Variables column should read as follows (correction in **bold**):

**Table 4:** Regression Results

Variables	(1) Difference between Final and Presenting VA	(2) Difference between Final and Presenting VA	(3) Difference between Final and Presenting VA	(4) Difference between Final and Presenting VA
				
On anticoagulant	–0.866∗	–0.711∗		
	(0.256)	(0.225)		
On antiplatelet			–0.426‡	–0.692∗
			(0.247)	(0.194)
Male sex	0.0373	–0.0438	0.330	0.313
	(0.287)	(0.280)	(0.256)	(0.216)
Age	–0.00926	–0.0165	0.00883	–0.00296
	(0.0146)	(0.0122)	(0.0138)	(0.00973)
Anti-VEGF	0.336	0.487	0.393	0.246
	(0.374)	(0.346)	(0.325)	(0.271)
Vitrectomy	0.431‡	0.312	0.155	–0.131
	(0.258)	(0.262)	(0.255)	(0.195)
Pneumatic displacement	–0.0741	–0.218	–0.163	–1.026∗
	(0.457)	(0.442)	(0.497)	(0.381)
Cataract surgery after SMH	–1.101∗	–0.924∗	–0.431	–0.890∗
	(0.308)	(0.300)	(0.273)	(0.234)
Initial VA	–0.528∗	–0.691∗	–0.570∗	–0.744∗
	(0.163)	(0.155)	(0.163)	(0.155)
Time to presentation compared with <7 d				
7–14 d	0.0968∗∗	0.0235	0.0548	0.0286
	(0.0490)	(0.0442)	(0.0498)	(0.0373)
15–30 d	0.468	–0.104	–0.0952	–0.151
	(0.340)	(0.313)	(0.314)	(0.229)
>30 d	0.537	0.473	0.336	0.583
	(0.409)	(0.361)	(0.481)	(0.451)
Time followed	–0.139	–0.788†	–0.0201	–0.523†
	(0.371)	(0.371)	(0.332)	(0.257)
SMH size compared with 1–3 DD				
**4–5** DD		1.517∗		2.200∗
		(0.485)		(0.335)
>5 DD		1.451∗		1.737∗
		(0.366)		(0.255)
SMH thickness (microns)		–0.000606		–0.000992∗
		(0.000458)		(0.000330)
Constant	1.074	2.187‡	–0.206	1.780‡
	(1.340)	(1.131)	(1.310)	(0.990)
Number of eyes	42	30	60	46
Number of subjects	40	29	59	45

DD = disc diameter; SMH = submacular hemorrhage; VA = visual acuity.

Regression results provided using a multivariate generalized estimating equation model to account for using both eyes of patients with bilateral submacular hemorrhage. Standard errors in parentheses.

∗ *P* < 0.01.

† *P* < 0.05.

‡ *P* < 0.1

“SMH size compared with 1-3 DD **4-5 DD**”

In Supplemental Tables 7 and 8, the first row of the first column under “Difference in Final and Presentation VA” should read as follows (correction in **bold**):

“On an **antiplatelet**”

**Figure:**
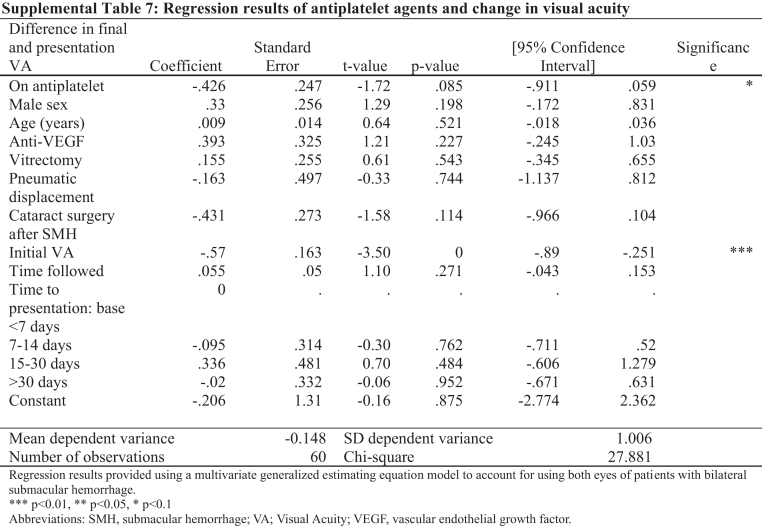

**Figure:**