# Supplementary material for: Oral Antithrombotic Medication Is Associated with Improved Visual Outcomes in Eyes with Submacular Hemorrhage from Wet Age-Related Macular Degeneration
Source: Ophthalmol Sci. 2025 Apr 14;5(5):100796. doi: 10.1016/j.xops.2025.100796 (PMC12143626; doi:10.1016/j.xops.2025.100796)
Supplement: Table S5 [file mmc1.pdf]

Supplemental Table 5. Regression results of anticoagulants and difference in visual acuity

Difference in final and presentation VA	Coefficient	Standard error	t-value	p-value	[95% Confidence	Interval]	Significance
On an anticoagulant	-.866	.256	-3.38	.001	-1.368	-.364	***
Male sex	.037	.287	0.13	.897	-.525	.6	
Age (years)	-.009	.015	-0.63	.527	-.038	.019	
Anti-VEGF	.336	.374	0.90	.369	-.398	1.07	
Vitrectomy	.431	.258	1.67	.094	-.074	.936	*
Pneumatic displacement	-.074	.457	-0.16	.871	-.97	.822	
Cataract surgery after SMH	-1.101	.308	-3.57	<0.001	-1.705	-.497	***
Initial VA	-.528	.163	-3.23	.001	-.848	-.208	***
Time followed	.097	.049	1.98	.048	.001	.193	**
Time to presentation: base <7 days	0	.	.	.	.	.	
7-14 days	.468	.34	1.38	.168	-.198	1.135	
15-30 days	.537	.409	1.32	.188	-.263	1.338	
>30 days	-.139	.371	-0.38	.708	-.866	.588	
Constant	1.074	1.34	0.80	.423	-1.552	3.7	

Mean dependent variance	-0.284	SD dependent variance	1.018
Number of observations	42	Chi-square	49.181

Regression results provided using a multivariate generalized estimating equation model to account for using both eyes of patients with bilateral submacular hemorrhage.

*** p<0.01, ** p<0.05, * p<0.1

Abbreviations: SMH, submacular hemorrhage; VA; Visual Acuity; VEGF, vascular endothelial growth factor.
