# Supplementary material for: Oral Antithrombotic Medication Is Associated with Improved Visual Outcomes in Eyes with Submacular Hemorrhage from Wet Age-Related Macular Degeneration
Source: Ophthalmol Sci. 2025 Apr 14;5(5):100796. doi: 10.1016/j.xops.2025.100796 (PMC12143626; doi:10.1016/j.xops.2025.100796)
Supplement: Table S6 [file mmc2.pdf]

Supplemental Table 6. Regression results of anticoagulants and change in visual acuity controlling for SMH size

Difference in final and presentation VA	Coefficient	Standard Error	t-value	p-value	[95% Confidence	Interval]	Significance
On an anticoagulant	-.711	.225	-3.15	.002	-1.152	-.269	***
Male sex	-.044	.28	-0.16	.876	-.592	.504	
Age (years)	-.017	.012	-1.35	.177	-.04	.007	
Anti-VEGF	.487	.346	1.41	.158	-.19	1.165	
Vitrectomy	.312	.262	1.19	.234	-.202	.826	
Pneumatic displacement	-.218	.442	-0.49	.622	-1.084	.648	
Cataract surgery after SMH	-.924	.3	-3.08	.002	-1.512	-.336	***
Initial VA	-.691	.155	-4.45	0	-.996	-.387	***
Time followed	.024	.044	0.53	.594	-.063	.11	
Time to presentation: base <7 days	0	.	.	.	.	.	
7-14 days	-.104	.313	-0.33	.741	-.718	.511	
15-30 days	.473	.361	1.31	.19	-.235	1.181	
>30 days	-.788	.371	-2.13	.034	-1.516	-.061	**
SMH Size: base 0-3 DD	0	.	.	.	.	.	
4-5 DD	1.517	.485	3.13	.002	.566	2.468	***
>5 DD	1.451	.366	3.96	0	.732	2.169	***
SMH thickness (μm)	-.001	0	-1.32	.185	-.002	0	
Constant	2.187	1.131	1.93	.053	-.03	4.403	*

Mean dependent variance	-0.040	SD dependent variance	0.961
Number of observations	30	Chi-square	97.107

Regression results provided using a multivariate generalized estimating equation model to account for using both eyes of patients with bilateral submacular hemorrhage.
*** p<0.01, ** p<0.05, * p<0.1
Abbreviations: DD, disc diameters; SMH, submacular hemorrhage; VA; Visual Acuity; VEGF, vascular endothelial growth factor.
