# Supplementary material for: Oral Antithrombotic Medication Is Associated with Improved Visual Outcomes in Eyes with Submacular Hemorrhage from Wet Age-Related Macular Degeneration
Source: Ophthalmol Sci. 2025 Apr 14;5(5):100796. doi: 10.1016/j.xops.2025.100796 (PMC12143626; doi:10.1016/j.xops.2025.100796)
Supplement: Table S7 [file mmc3.pdf]

Supplemental Table 7. Regression results of antiplatelet agents and change in visual acuity

Difference in final and presentation VA	Coefficient	Standard Error	t-value	p-value	[95% Confidence Interval]		Significance
On an anticoagulant	-.426	.247	-1.72	.085	-.911	.059	*
Male sex	.33	.256	1.29	.198	-.172	.831	
Age (years)	.009	.014	0.64	.521	-.018	.036	
Anti-VEGF	.393	.325	1.21	.227	-.245	1.03	
Vitrectomy	.155	.255	0.61	.543	-.345	.655	
Pneumatic displacement	-.163	.497	-0.33	.744	-1.137	.812	
Cataract surgery after SMH	-.431	.273	-1.58	.114	-.966	.104	
Initial VA	-.57	.163	-3.50	0	-.89	-.251	***
Time followed	.055	.05	1.10	.271	-.043	.153	
Time to presentation: base <7 days	0	.	.	.	.	.	
7-14 days	-.095	.314	-0.30	.762	-.711	.52	
15-30 days	.336	.481	0.70	.484	-.606	1.279	
>30 days	-.02	.332	-0.06	.952	-.671	.631	
Constant	-.206	1.31	-0.16	.875	-2.774	2.362	

Mean dependent variance	-0.148	SD dependent variance	1.006
Number of observations	60	Chi-square	27.881

Regression results provided using a multivariate generalized estimating equation model to account for using both eyes of patients with bilateral submacular hemorrhage.
*** p<0.01, ** p<0.05, * p<0.1
Abbreviations: SMH, submacular hemorrhage; VA; Visual Acuity; VEGF, vascular endothelial growth factor.
