# Supplementary material for: Oral Antithrombotic Medication Is Associated with Improved Visual Outcomes in Eyes with Submacular Hemorrhage from Wet Age-Related Macular Degeneration
Source: Ophthalmol Sci. 2025 Apr 14;5(5):100796. doi: 10.1016/j.xops.2025.100796 (PMC12143626; doi:10.1016/j.xops.2025.100796)
Supplement: Table S8 [file mmc4.pdf]

Supplemental Table 8. Regression results of antiplatelet agents and change in visual acuity controlling for SMH size

Difference in final and presentation VA	Coefficient	Standard Error	t-value	p-value	[95% Confidence	Interval]	Significance
On an anticoagulant	-.692	.194	-3.56	0	-1.073	-.312	***
Male sex	.313	.216	1.45	.147	-.11	.736	
Age (years)	-.003	.01	-0.30	.76	-.022	.016	
Anti-VEGF	.246	.271	0.91	.365	-.286	.777	
Vitrectomy	-.131	.195	-0.67	.503	-.513	.252	
Pneumatic displacement	-1.026	.381	-2.69	.007	-1.773	-.279	***
Cataract surgery after SMH	-.89	.234	-3.81	0	-1.348	-.432	***
Initial VA	-.744	.155	-4.78	0	-1.048	-.439	***
Time followed	.029	.037	0.77	.444	-.045	.102	
Time to presentation: base <7 days	0	.	.	.	.	.	
7-14 days	-.151	.229	-0.66	.509	-.6	.297	
15-30 days	.583	.451	1.29	.196	-.301	1.467	
>30 days	-.523	.257	-2.04	.041	-1.027	-.02	**
SMH Size: base 0-3 DD	0	.	.	.	.	.	
4-5 DD	2.2	.335	6.57	0	1.544	2.857	***
>5 DD	1.737	.255	6.80	0	1.237	2.238	***
SMH thickness (μm)	-.001	0	-3.01	.003	-.002	0	***
Constant	1.78	.99	1.80	.072	-.161	3.721	*

Mean dependent variance	-0.024	SD dependent variance	1.007
Number of observations	46	Chi-square	116.272

Regression results provided using a multivariate generalized estimating equation model to account for using both eyes of patients with bilateral submacular hemorrhage.

*** p<0.01, ** p<0.05, * p<0.1

Abbreviations: DD, disc diameters; SMH, submacular hemorrhage; VA; Visual Acuity; VEGF, vascular endothelial growth factor.
